# Viral Reactivation in Multiple Myeloma Patients Receiving Anti-BCMA Chimeric Antigen Receptor T-Cell Therapy

**DOI:** 10.3390/ijms27073113

**Published:** 2026-03-30

**Authors:** Ido Cohen, Eyal Lebel, Sigal Grisariu, Batia Avni, Shlomit Kfir-Erenfeld, Nathalie Asherie, Eran Zimran, Vladimir Vainstein, Miri Assayag, Tatyana Dubnikov Sharon, Rivka Alexander-Shani, Nomi Bessig, Alaa Shehadeh, Aseel Ishtay, Miriam Schlossberg, Marjorie Pick, Moshe E. Gatt, Tali Bdolah-Abram, Polina Stepensky, Shlomo Elias

**Affiliations:** 1 Faculty of Medicine, Hebrew University of Jerusalem, Jerusalem 91120, Israel; ido.cohen7@mail.huji.ac.il (I.C.);; 2 Department of Hematology, Hadassah Medical Center, Faculty of Medicine, Hebrew University of Jerusalem, Jerusalem 91120, Israelrmoshg@hadassah.org.il (M.E.G.); 3 Department of Bone Marrow Transplantation and Cancer Immunotherapy, Hadassah Medical Center, Faculty of Medicine, Hebrew University of Jerusalem, Jerusalem 91120, Israelnathaliea@hadassah.org.il (N.A.);

**Keywords:** multiple myeloma, chimeric antigen receptor T cell, CAR-T-cell therapy, BCMA, cytomegalovirus, Epstein–Barr virus, adenovirus, viral reactivation, B cell lymphoma

## Abstract

Chimeric antigen receptor T (CAR-T) cell therapy has become a standard of care for many hematological malignancies, and has significantly transformed treatment outcomes. However, CAR-T therapy is associated with specific toxicities, including infections. Although the anti-CD19 CAR-T risks are well-characterized, infectious complications following B-cell maturation antigen (BCMA)-directed CAR-T in multiple myeloma (MM) remain under-researched. In this study, we evaluated the incidence and clinical impact of cytomegalovirus (CMV), Epstein–Barr virus (EBV), and adenovirus (ADV) reactivations in 75 patients receiving anti-BCMA CAR-T for MM, and compared them to 60 patients receiving commercial anti-CD19 CAR-T for B-cell lymphoma (BCL). The viral reactivation rates were 20% for CMV and 8% for EBV in the MM group, vs. 31.7% and 3%, respectively, in the BCL group. No ADV reactivations were seen in either cohort. Most of the CMV reactivations (87% in the MM cohort and 68.5% in the BCL cohort) were asymptomatic and clinically insignificant, and had no impact on progression-free survival (PFS) or overall mortality. Overall, these findings suggest that although CMV and EBV reactivations are relatively common after anti-BCMA CAR-T, they are rarely associated with meaningful disease, and the risks do not exceed those of CD19-directed therapy. Thus, routine pre-emptive screening for these viruses may be unwarranted in asymptomatic patients.

## 1. Introduction

Chimeric antigen receptor T-cell (CAR-T) therapy has transformed immunotherapeutic approaches for relapsed or refractory (R/R) MM. Ongoing research in frontline settings suggests that this new therapeutic option can be utilized as early as the second line [1,2]. However, CAR-T therapy poses a significant risk for a range of toxicities, including cytopenias, hypogammaglobulinemia, inflammatory toxicities such as cytokine release syndrome (CRS) and immune effector cell-associated neurotoxicity syndrome (ICANS). Immunosuppression constitutes yet another toxicity that can result from the underlying disease, previous lines of therapy, and/or the treatment itself through off-tumor plasma cell depletion by anti-BCMA CART cells and lymphodepleting chemotherapy administered before infusion. Collectively, these factors increase the risk of infection, including viral reactivations subsequent to CAR-T therapy targeting BCMA, thus underscoring the need for careful monitoring and supportive care.

Few works have documented viral reactivations after CAR-T therapy. Most have been retrospective studies on anti-CD19 CAR-T therapy with small sample sizes. In these anti-CD19 studies, the CMV reactivation rates were approximately 20–25% [3,4,5]. There are only a handful of studies specifically on anti-BCMA CAR-T therapy. In one study conducted at a single center in China, 61 MM patients treated with anti-BCMA CAR-T (two different products) were evaluated for viral infections [6]. Viral reactivations were reported in 24.5% of patients and included CMV, EBV, hepatitis B virus (HBV), herpes zoster, and COVID-19. CMV reactivation occurred in 10% of the patients, all within six months post-infusion. The patients received pre-emptive treatment with ganciclovir and immunoglobulins based on viral load monitoring. However, the authors did not report the viral load thresholds used to guide treatment. CMV reactivation was more common in patients treated with glucocorticoids, whereas EBV reactivation (7% of the total study population) appeared less frequently in this subgroup.

In 2024, the American Society for Transplantation and Cellular Therapy (ASTCT) published updated guidelines and recommendations for treatment and follow-up after CAR-T therapy [7]. The panel recommended that weekly CMV surveillance should be considered during the first 2 to 6 weeks post-CAR-T infusion in patients with: (1) a previous history of CMV reactivation, (2) more than three days of corticosteroid use, (3) receipt of anti-BCMA CAR-T products, or (4) multiple lines of chemotherapy before CAR-T cell therapy. Routine polymerase chain reaction (PCR) surveillance for EBV and adenovirus was not recommended.

The dearth of studies on viral reactivation after CAR-T therapy, and anti-BCMA CAR-T in particular, also clearly points to the need for more comprehensive research, given that most studies are retrospective, involve small cohorts, and lack sufficient follow-up to evaluate the incidence and clinical significance of viral reactivations such as CMV, EBV, and ADV. HBI0101 is a novel, second-generation optimized anti-BCMA, academic CAR-T product developed at Hadassah Medical Center, Israel. This point of care CAR-T product was specifically developed to respond to current economic and logistical constraints and to ensure adequate access for all patients in need of next-generation treatment.

This therapy has demonstrated encouraging outcomes, with an overall response rate of 92% and a complete response (CR)/stringent CR rate of 55%. Two studies found a median progression-free survival (PFS) of 11.6 months after a median follow-up of one year, thus showing its clinical potential [8,9]. The current study was designed to address current knowledge gaps by investigating the incidence and clinical significance of viral reactivations in MM patients treated with anti-BCMA CAR-T therapy compared to a control cohort of lymphoma patients receiving anti-CD19 CAR-T therapy.

## 2. Results

### 2.1. Patient Characteristics

A total of 135 patients were evaluated. Seventy-five were MM patients treated with anti-BCMA CAR-T (HBI0101), and 60 were BCL patients treated with commercial anti-CD19 CAR-T. The latter cohort comprised 50 patients with diffuse large BCL (83.3%), five with high grade BCL (8.3%), four with primary mediastinal BCL (6.6%), and one with mantle cell lymphoma (1.7%). The median number of previous treatment lines was four (range: 1–13) across all patients, five (range: 3–13) in the MM cohort, and three (range: 1–13) in the BCL cohort. The patients’ characteristics are detailed in Table 1.

### 2.2. Viral Reactivations

In the MM cohort, 15 out of the 75 (20%) patients experienced CMV reactivation (rCMV) compared to 19 out of the 60 (31.7%) in the BCL cohort (*p* = 0.12). Of these reactivations, 2 out of 15 (13%) in the MM cohort and 6 out of 19 (31.5%) in the BCL cohort were clinically significant (CS). In the MM cohort, the median and mean time to rCMV were 70 and 176.8 days, respectively, compared to 241.1 and 96 days in the BCL cohort (*p* = 1 and *p* = 0.53, respectively). The rCMV time range was 10–769 days in the MM cohort and 7–928 days in the BCL cohort. The median time to clinically significant CMV reactivation (CS-rCMV) was 145.5 days post-infusion, with a mean of 187.2 days. Notably, both MM patients and five out of the six BCL patients with CS-rCMV experienced disease progression prior to reactivation; the exception was one patient who developed CS-rCMV 12 days after infusion. Out of all the CS-rCMV patients, regardless of relapse status, all but two B-cell lymphoma patients were administered additional anti-cancer treatment after CAR-T infusion, during or shortly before the time of the CMV reactivation. This included chemotherapy (four patients), immune checkpoint inhibitors (two patients), and bispecific antibodies (three patients) (Table 2). Of the eight patients with CS-rCMV, three patients (one MM and two BCL patients) died from concurrent sepsis secondary to another infection (other than the CMV) or disease progression.

The median blood CMV PCR viral load in the MM patients with viral reactivation was 695 copies/mL, with 47% of the patients exceeding 1000 copies/mL. In the BCL cohort, the median viral load was 733 copies/mL, with 47% exceeding 1000 copies/mL. None of the patients with CS-rCMV infections had a blood CMV PCR viral load exceeding 10,000 copies/mL, and only three out of eight (37.5%) exceeded 5000 copies/mL, although in some patients the viral load detected by tissue PCR was substantially higher.

Most CMV reactivations occurred within the first six months post-infusion in both cohorts; specifically, 66.6% in the MM cohort and 63.1% in the BCL cohort, with a median time to CMV reactivation in the MM cohort of 70 days (range 10–769) compared to 96 (range 7–916) in the BCL cohort, respectively. A substantial proportion of these reactivations took place early, with 6 out of the 15 (40%) rCMV cases in the MM cohort and 8 out of 19 (44.4%) in the BCL cohort occurring within the first 45 days post-infusion. In the BCL cohort, CS-rCMV also mainly occurred within the first six months, with 4 out of the 6 cases (66.7%) presenting in this timeframe. In the MM cohort, of the two CS-rCMV cases observed, one occurred within six months and the other at seven months post-infusion. EBV reactivation occurred in 6 of the 75 MM patients (8%) and 2 of the 60 (3%) BCL patients (*p* = 0.25). No ADV reactivation was detected in either group.

### 2.3. Associations Between rCMV and Patients’ Characteristics in the MM Cohort

No associations were found between rCMV and the patients’ baseline or disease characteristics, including the number of previous therapy lines (*p* = 0.92), a previous autologous stem cell hematopoietic transplant (ASCT) (*p* = 0.85), previous reactivation or viral carrier status (*p* = 0.69), R-ISS stage at infusion (*p* = 0.53), high risk cytogenetics (*p* = 0.07), ECOG (*p* = 0.07) or other features (Table 3). By contrast, high ferritin levels at the time of infusion were found to have a statistically significant association with rCMV: every 10 ng/mL increase in ferritin corresponded to a 1.01-fold increase in the risk of CMV reactivation (odds ratio 1.01, 95% CI 1.00–1.02).

### 2.4. Associations Between rCMV and Other CAR-T-Related Toxicities in the MM Cohort

No association was found between rCMV and other CART toxicities, including CRS (*p* = 0.57), ICANS (*p* = 1), febrile neutropenia (*p* = 0.18), corticosteroid use for more than three days (*p* = 0.99), lymphopenia 1–28 days post-infusion or other toxicities (Table 4). However, patients with rCMV exhibited a significantly lower likelihood of early bacteremia (up to day +28) compared to non-reactivated patients, with an odds ratio (OR) of 14.75 (95% CI 1.4–154.1, *p* = 0.02 Table 4).

### 2.5. Associations Between Viral Reactivations and Survival

**Figure 1:**
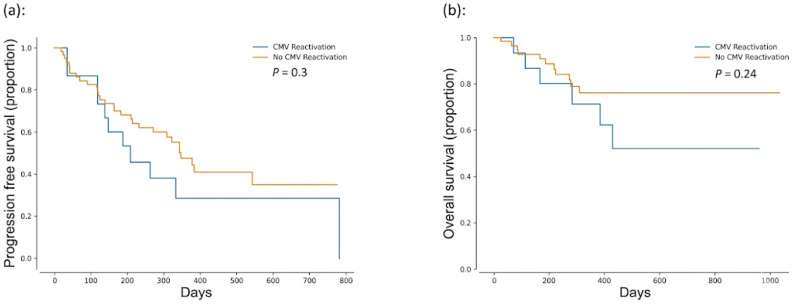
Progression-free and overall survival outcomes after anti-BCMA CART as a function of cytomegalovirus (CMV) reactivation status. (**a**) Progression-free survival in patients with and without CMV reactivation; (**b**) overall survival in patients with and without CMV reactivation.

In the MM cohort, patients with rCMV had a numerically lower progression-free survival (PFS) and overall survival (OS) rate than patients without rCMV; however, these differences were not statistically significant (median PFS 208 vs. 342 days, *p* = 0.30; median OS 623 vs. 825 days, *p* = 0.24; Figure 1a,b). A similar pattern was observed in the BCL cohort, with no significant differences in PFS (median 391.7 vs. 470.8 days, *p* = 0.82) or OS (median 670.6 vs. 710 days, *p* = 0.96) between patients with and without rCMV.

In both cohorts, time to rCMV was not associated with change in PFS (MM: *p* = 0.86; BCL: *p* = 0.65) or OS (MM: *p* = 0.36; BCL: *p* = 0.14). These findings were also found for the pooled analysis of all patients, in which time to rCMV had no impact on PFS or OS (*p* = 0.65 and *p* = 0.09, respectively). The small number of patients with clinically significant rCMV (CS-rCMV) precluded a meaningful assessment of its effect on survival outcomes. Similarly, EBV reactivation was not associated with PFS or OS, although the low incidence prevents drawing definitive conclusions.

## 3. Discussion

This study investigated the incidence and clinical significance of viral reactivations in patients treated with CAR-T-cell therapies targeting either BCMA (for MM) or CD19 (for BCL), using a prospective cohort for MM and a retrospectively collected control group for BCL. There was a high rate of CMV reactivations in both cohorts. However, the vast majority were clinically insignificant, with 87% in the MM group and 68.5% in the BCL group presenting as self-limiting infections that resolved without the need for antiviral treatment or hospitalization. In particular, reactivations of CMV and EBV had no detectable impact on PFS or OS in either cohort, and adenovirus reactivation was not observed.

Infections are a common and serious complication in recipients of CAR-T-cell therapy that contribute significantly to both morbidity and mortality. According to recent guidelines published by the European Society for Blood and Marrow Transplantation (EBMT) and the European Hematology Association (EHA) as well as ASTCT monitoring for CMV after CAR-T therapy should be considered in patients deemed as high risk [10]. Specifically, this includes patients with histories of allogeneic hematopoietic cell transplantation, patients who received high-dose or prolonged corticosteroid therapy, patients who had received multiple previous chemotherapy lines and/or patients receiving anti-BCMA CAR-T. Routine surveillance for EBV, human herpes virus 6, and adenovirus is not recommended. Both sets of guidelines (EBMT/EHA and ASTCT) nevertheless acknowledge the scant available data on the clinical impact of CMV reactivation, especially with anti-BCMA CAR-T, and the importance of optimal management strategies.

Previous studies have reported CMV reactivation rates of up to 25% after CAR-T-cell therapy [3,4]. One study indicated a ~10% lower incidence in patients treated with BCMA-directed CAR-T cells [6]. Across studies, rCMV has typically been found to occur within the first six months after infusion, but in some cases earlier. Factors such as BCMA-directed CAR-T and corticosteroid exposure for three or more days have been associated with increased risk, which is consistent with the ASTCT guidelines. However, most published cohorts have included heterogeneous CAR-T products, which makes it difficult to draw conclusions specific to anti-BCMA therapy [4,5]. In the Kampouri et al. [4] study, for example, CS-rCMV, defined as CMV viremia ≥ 150 IU/mL (which is the institutional threshold for initiating pre-emptive treatment), occurred in 9% of the patients. Although overall survival was lower in patients with reactivation, the difference did not reach statistical significance. In our study, the univariable Cox regression analysis indicated that CMV reactivation, including CS-rCMV, showed a trend toward increased overall mortality, although this did not reach statistical significance. Li et al. [5] identified anti-BCMA CAR-T products (*p* = 0.009), CRS grade ≥ 3 (*p* < 0.001), receipt of high doses of steroids (*p* = 0.017) and elevated IL-6 levels (*p* = 0.003) as risk factors for rCMV. However, in their subgroup analyses, IL-6 did not retain its statistical significance when stratified by CAR-T target or underlying diagnosis.

Crucially, both Kampouri and Li et al. [4,5] examined relatively small samples of patients receiving anti-BCMA CAR-T therapy. Li et al. [5] reported no cases of clinical CMV disease, and in Kampouri et al. [4], the threshold for defining CS-rCMV (150 IU/mL, ≈165 copies/mL) was comparatively low. In our study, nearly all patients with detectable CMV PCR (31/37; 83.78%) had viral loads exceeding 165 copies/mL.

Our findings align with the literature on CMV reactivation rates but also reveal key differences [7,10]. Viral reactivations post-anti-BCMA CAR-T were common but lower than post-anti-CD19 CAR-T, contrary to other published data. Bear in mind, however, that lymphodepletion protocols between the two CAR-T therapies differ slightly, mostly in their doses. In addition, whereas most of the reactivations occurred within the first six months post-infusion (72.2% in the MM cohort and 65% in the BCL cohort), not all cases followed this pattern, unlike findings reported in some previous studies. Furthermore, the occurrence of rCMV did not demonstrate a statistically significant effect on PFS or OS, within each cohort or in the overall study population. Factors previously identified as risks for CMV reactivation such as steroid use for more than three days, number of previous therapies, and ASCT were not associated with higher rates of CMV reactivation in our cohort. Disease characteristics, including cytogenetics, and ISS/R-ISS, also appeared to have no effect on the risk of viral reactivations. Similarly, the time to reactivation, occurrence of CRS, ICANS, or the use of post-infusion immunosuppression (regardless of the number of days), did not influence reactivation rates. Interestingly, in the MM cohort, early bacteremia (days 1–28 post-infusion) was associated with a trend toward reduced risk of CMV reactivation, with an odds ratio of 14.75. Conversely, elevated ferritin levels at infusion were linked to an increased risk of CMV reactivation, with an odds ratio of 1.01 per 10 ng/mL increase in plasma ferritin levels.

Although both early bacteremia and elevated ferritin signal a heightened inflammatory state, they have opposite effects on CMV reactivation. The acute pro-inflammatory surge associated with bacteremia may create a hostile “bystander” environment that suppresses viral replication. Conversely, elevated ferritin likely reflects a background of chronic inflammation or immune dysregulation, which typically fails to control latency and instead creates a favorable environment for viral reactivation. Ferritin may therefore contribute to the risk stratification of CMV reactivation in high-risk patients, although additional prospective studies are needed to validate these findings. Most importantly, seven out of the eight CS-rCMV cases in the current study occurred after disease progression and during treatment with additional therapies, such as bispecific antibodies or chemotherapy. This suggests that CMV reactivation may be more closely related to subsequent lines of immunosuppressive treatment than to the CAR T-cell therapy itself. Previous reports in newly diagnosed MM patients treated with novel agents have found CMV reactivation rates of approximately 20%, thus highlighting the baseline immunocompromised state of MM patients [11]. Patients experiencing disease progression after CAR-T therapy represent a particularly vulnerable population, because undiagnosed CMV reactivation can severely delay the initiation of urgent subsequent therapies. Although universal screening guidelines require further prospective validation, our findings suggest that heightened clinical vigilance is warranted and that targeted CMV monitoring in patients exhibiting high-risk features such as those described in the literature and in this study may be needed to prevent detrimental delays in salvage treatment. Overall, the CMV reactivations were largely benign and did not impact clinical outcomes, suggesting that routine surveillance may be unnecessary and that a more targeted approach focusing on symptomatic individuals may be more appropriate.

This study has several limitations. The total sample size was small. This was reflected in the small number of CS-rCMV cases, which limited the statistical power to evaluate associations with clinical outcomes such as OS or PFS. In addition, this was a single-center study with a single anti-BCMA CAR-T product, which may preclude the generalizability of the findings. Finally, most cases of CS-rCMV occurred after disease progression and during treatment with additional therapies, thus making it difficult to determine whether the reactivation was directly related to CAR-T-cell therapy.

## 4. Materials and Methods

This prospective study included all MM patients enrolled in the NCT04720313 phase 1a/b study with HBI0101 CAR-T from study initiation in February 2021 up to July 2023 who were eventually infused with the CAR-T product. The key inclusion criteria for NCT04720313 were age ≥ 18, at least three previous therapy lines (including proteasome inhibitors, immunomodulatory drugs, and an anti-CD38 antibody), and measurable disease at screening [8]. The complete inclusion and exclusion criteria are detailed in the NCT04720313 study report [8]. The BCL cohort, collected retrospectively, included all patients in the Hadassah Medical Center between December 2019 and June 2023 who received anti-CD19 CAR-T: axicabtagene ciloleucel (axi-cel; Yescarta) Kite Pharma (Gilead), El Segundo, CA, USA. tisagenlecleucel (tisa-cel; Kymriah) Novartis, Morris Plains, NJ, USA. and brexucabtagene autoleucel (Tecartus; brexu-cel) Kite Pharma (Gilead), El Segundo, CA, USA. Both groups underwent lymphodepletion with fludarabine and cyclophosphamide on days −5 to −3 before infusion and adjustments based on renal function. In the case of creatinine clearance of less than 30 mL/min, bendamustine 90 mg/m^2^ for 2 days was administered as an alternative to fludarabine and cyclophosphamide. All patients received antiviral prophylaxis with acyclovir 800 mg twice a day, without any CMV prophylaxis.

### 4.1. Data Collection

All patients were hospitalized for lymphodepleting chemotherapy and CAR-T infusion and remained hospitalized for close monitoring and management, typically up to 14 days post-infusion, although discharge could occur earlier based on clinical stability. Both cohorts underwent disease response evaluation at day +30, based on the International Myeloma Working Group (IMWG) criteria for MM patients and the Lugano Criteria for BCL patients [12,13]. The MM cohort had additional assessments at days +60, +120, and +180 post-infusion, followed by evaluations every three months. The BCL cohort was assessed at day +180 and subsequently every three months. CRS and ICANS were graded according to the 2019 ASTCT criteria [14]. All data collection and analyses were conducted in accordance with the Declaration of Helsinki and received approval from the Hadassah Medical Center Institutional Review Board (0090-20-HMO) and from the Israel Ministry of Health (202016845).

### 4.2. Viral Tests

Viral reactivation was defined as a PCR load >100 copies/mL, and CS reactivation was defined as a need for antiviral treatment (as determined by the treating physician) or hospitalization. Viral serology for CMV, EBV and ADV, as well as HBV and hepatitis C virus (HCV), was assessed pre-infusion to determine carrier status. Viral PCR testing was also conducted during the initial hospitalization and at each follow-up visit. For the MM cohort, PCR testing was conducted at days +7–14, +30, +60, +120, +180, and then every six months. In the BCL cohort, testing occurred once between days +7–14 and at day +30, after which it was performed at the discretion of the treating physician. In the case of suspected CMV disease, a targeted biopsy of the affected organ was conducted to identify CMV in the tissue using PCR and immunohistochemistry.

### 4.3. Statistical Analysis

Kaplan–Meier curves were utilized to characterize viral reactivations, CS infections rates, and time to infection. Logistic regression was employed to identify significant risk factors associated with viral reactivations and CS infections in the study group. For the quantitative variables, either Student’s *t*-test or the Mann–Whitney test was utilized. Categorical variables were analyzed using a chi-square or Fisher’s exact test, as appropriate, prior to running the logistic regression model. Cox regression was employed to analyze the effect of time to reactivation on disease outcome. All tests applied were two-tailed, and a *p*-value of 0.05 or less was considered statistically significant. Statistical analyses were implemented with the SPSS software, version 31.0.0, IBM Corporation, Armonk, NY, USA.

## 5. Conclusions

Viral reactivations are a common complication after CAR-T therapy, and CMV in particular, which manifested in 20% of the MM patients receiving anti-BCMA CAR-T. In the current study, these reactivations generally lacked clinical significance, as evidenced by the absence of associations with increased mortality or reduced PFS, suggesting that routine testing or preventive treatment may be unnecessary. Future research should focus on larger, multi-center cohorts to validate these findings, investigate the relationships between the timing of reactivation and clinical outcomes, and explore the biomarkers predictive of reactivation risk. Optimizing antiviral prophylaxis and defining thresholds for intervention remain critical for improving CAR-T therapy safety profiles.

## Figures and Tables

**Table 1:** Baseline patient and disease characteristics prior to CAR-T infusion.

Characteristic	Total	Multiple Myeloma	B Cell Lymphoma	*p* Value ^a^
Gender				0.54
Male, n (%)	67 (49.6%)	39 (52%)	28 (46.7%)	
Female, n (%)	68 (50.4%)	36 (48%)	32 (53.3%)	
Age at Diagnosis, Median (range)—years	58 (27–87)	58 (35–80)	62.5 (27–87)	0.34
Time from diagnosis to infusion, Mean ± SD—months	60.3 ± 65.7	74.1 ± 55.9	42.5 ± 73.3	0
ECOG at enrollment, n (%)				0.055
0	24 (17.8%)	9 (12%)	15 (25%)	
1	47 (34.8%)	25 (33.3%)	22 (36.7%)	
2	60 (44.4%)	40 (53.3%)	20 (33.3%)	
3	3 (2.2%)	1 (1.3%)	2 (3.3%)	
4	1 (0.7%)	0 (0%)	1 (1.7%)	
Past viral reactivation/carrier, n (% of evaluable patients)				0.3
None	125 (93.2%)	68 (90.7%)	57 (96.6%)	
HBV	6 (4.4%)	4 (5.3%)	2 (3.4%)	
HCV	2 (1.5)	2 (2.7%)	0 (0%)	
CMV and EBV	1 (0.7%)	1 (1.3%)	0 (0%)	
Number of previous therapy lines, median (range)	4 (1–13)	5 (3–13)	3 (1–11)	0
Previous ASCT, n (% from evaluable)	56 (41.5%)	52 (69.3%)	4 (6.7%)	0
CAR-T Product, n (%)				N/A
anti-BCMA- HBI0101	75 (55.6%)	75 (100%)	0 (0%)	
Axicabtagene ciloleucel	7 (5.2%)	0 (0%)	7 (11.7%)	
Tisagenlecleucel	52 (38.5%)	0 (0%)	52 (86.6%)	
Brexucabtagene autoleucel	1 (0.7%)	0 (0%)	1 (1.7%)	
Stage at enrollment ^b^, n (% of evaluable)				N/A
0	N/A	N/A	16 (28%)	
1	N/A	12 (20.6%)	10 (17.5%)	
2	N/A	39 (67.2%)	4 (7%)	
3	N/A	7 (12%)	13 (22.8%)	
4	N/A	N/A	14 (24.5%)	
CD4 count at lymphodepletion, Mean ± SD, cells/mm^3^	N/A	N/A	280.43 ± 212.45	N/A
CD8 count at lymphodepletion, Mean ± SD, cells/mm^3^	N/A	N/A	402.53 ± 357.52	N/A
IgG pre-infusion, Mean ± SD, mg/dL	933 ± 1298	1271 ± 1661	628 ± 232	0.93
IgM pre-infusion, Mean ± SD, mg/dL	35 ± 76	21 ± 35	53 ± 106	0
IgA pre-infusion, Mean ± SD, mg/dL	179 ± 415	228 ± 541	115 ± 89	0
CRP (at least 10 days prior to infusion), Mean ± SD, mg/L	4.5 ± 23.7	5.8 ± 31.3	3.0 ± 6.5	0.86
Ferritin (at least 10 days prior to infusion), Mean ± SD, µg/L	610 ± 1675	429 ± 520	824 ± 2409	0.13
WBC at lymphodepletion, Mean ± SD, 10^9^/L	4.5 ± 2.0	4.1 ± 1.6	4.9 ± 2.4	0.03
ANC at lymphodepletion, Mean ± SD, 10^9^/L	2.92 ± 1.5	2.6 ± 1.3	3.3 ± 1.8	0.01
ALC at lymphodepletion, Mean ± SD, 10^9^/L	0.8 ± 0.5	0.9 ± 0.5	0.8 ± 0.6	0.64

n: number; ECOG: Eastern Cooperative Oncology Group Performance Status; HBV: hepatitis B virus; HCV: hepatitis C virus; CMV: cytomegalovirus; EBV: Epstein–Barr virus; ASCT: autologous stem cell transplant; BCMA: B-cell maturation antigen; IgG: immunoglobulin G; IgM: immunoglobulin M; IgA: immunoglobulin A; CRP: C-reactive protein; WBC: white blood cells; ANC: Absolute Neutrophil Count; ALC: Absolute Lymphocyte Count; N/A: not applicable. ^a^ Statistical analysis implemented chi-square tests for categorical variables, and Fisher’s exact tests when appropriate. *t*-test was used for continuous variables. ^b^ The MM staging corresponds to the revised international staging system (R-ISS), BCL staging was based on the Ann Arbor scale.

**Table 2:** Clinical characteristics of patients with clinically significant CMV reactivation.

Pt. No.	1	2	3	4	5	6	7	8
Disease	MM	MM	BCL	BCL	BCL	BCL	BCL	BCL
CAR-T type	HBI0101	HBI0101	Tisagenlecleucel	Tisagenlecleucel	Tisagenlecleucel	Tisagenlecleucel	Axicabtagene ciloleucel	Tisagenlecleucel
Best response	PD	VGPR	PD	PD	PD	PD	CR	PD
Time to relapse (days)	34	147	68	45	56	30	90	42
Relapse preceded reactivation: Y/N	Y	Y	N	Y	Y	Y	Y	Y
Next treatment lines	Chemotherapy + bi-specific Ab	Chemotherapy + bi-specific Ab	none	Bi-specific Ab + gazayva	ICI + chemotherapy	ICI	chemotherapy	none
Time to reactivation (days)	91	214	12	109	197	597	182	96
Clinical CMV disease	colitis	colitis	fever	pneumonitis	pneumonitis	gastritis	colitis	general malaise
Blood CMV viral load by PCR (copies/mL)	6414	2887	5888	3950	413	1492	5429	1638
Tissue CMV viral load by PCR (copies/mL)	N/A	7802	N/A	200,600	1743	54,312	N/A	N/A
Reactivation treatment	none	valganciclovir	foscarnet, ganciclovir	ganciclovir	ganciclovir	ganciclovir	ganciclovir	valganciclovir
Death during CMV hospitalization	Y	N	N	Y	Y	N	N	N
Concurrent illness during CMV disease	sepsis		ICANS	CRS, sepsis	sepsis		Active T-cell lymphoma	
Cause of death	pneumonia			COVID-19	COVID-19			

MM: multiple myeloma; BCL: B cell lymphoma; CAR-T: chimeric antigen receptor T cell; CMV: Cytomegalovirus; VGPR: very good partial response; PD: progressive disease; PCR: polymerase chain reaction; ICI: immune checkpoint inhibitors; Ab: antibody; CRS: cytokine release syndrome; ICANS: Immune effector cell-associated neurotoxicity syndrome; N/A: not applicable.

**Table 3:** Associations between pre-infusion factors and CMV reactivation risk in multiple myeloma patients.

Factor	OR (95% CI), *p* Value
No. of previous lines	1.01 (0.79–1.28), 0.92
Time from Dx to Treatment (Months)	0.99, (0.98–1), 0.51
No. of viable CAR-T cells, 10^6^	0.99 (0.99–1), 0.47
CRP (at least 10 days prior to infusion), mg/L	1.02 (0.97–1.06), 0.38
Ferritin (at least 10 days prior to infusion), µg/L	1.01 ^a^ (1–1.02), 0.04
WBC at lymphodepletion, 10^9^/L	0.9 (0.62–1.31), 0.61
ANC at lymphodepletion, 10^9^/L	0.78 (0.48–1.28), 0.34
ALC at lymphodepletion, 10^9^/L	1.78 (0.62–5.07), 0.28
Prior ASCT	0.85 (0.25–2.86), 0.85
Prior anti-BCMA therapies	0.64 (0.07–5.78), 0.69
ECOG at enrolment	0.49 (0.22–1.06) 0.07
Past viral reactivation/carrier	0.64 (0.07–5.78), 0.69
CMV IgM pre-infusion status	1.28 (1.13–1.46), 0.34
R-ISS at enrolment	1.41 (0.47–4.27), 0.53
High risk cytogenetics	3.35 (0.93–12), 0.07

Dx: diagnosis; CAR-T: chimeric antigen receptor T cell; ECOG: Eastern Cooperative Oncology Group Performance Status; CMV: cytomegalovirus; ASCT: autologous stem cell transplant; BCMA: B-cell maturation antigen; R-ISS: revised international staging system; IgM: immunoglobulin M; CRP: C-reactive protein; WBC: white blood cells; ANC: Absolute Neutrophil Count; ALC: Absolute Lymphocyte Count. ^a^ Every 10 ng/mL increase in ferritin corresponded to a 1.01-fold increase in the risk of CMV reactivation.

**Table 4:** Associations between CAR-T toxicities and CMV reactivation risk in multiple myeloma patients (univariate analysis).

Factor	OR (95% CI), *p* Value
Neutropenia (Days 1–28 post- infusion)	0.21 (0.01–3.21), 0.26
Febrile neutropenia (Days 1–28 post-infusion)	2.48 (0.63–9.76), 0.18
Lymphopenia (Days 1–28 post-infusion)	^a^
elevated liver function tests (Days 1–28 post-infusion)	1.04 (0.54–1.99), 0.89
Early bacteremia (Days 1–28 post-infusion)	14.75 (1.41–154.15), 0.02
CRS (Days 1–28 post-infusion)	1.26 (1.12–1.43), 0.57
CRS grade	0.99 (0.4–2.41), 0.98
CRS start day	1.05 (0.88–1.25), 0.55
No. of Tocilizumab doses for CRS	0.78 (0.45–1.35), 0.38
Steroid use for CRS	0.37 (0.04–3.2), 0.67
≥4 days of steroids	0 (0–0), 0.99
Vasopressors for CRS	1.56 (0.27–9.02), 0.63
ICANS (Days 1–28 post-infusion)	0.79 (0.7–0.89), 1

CRS: cytokine release syndrome; ICANS: Immune effector cell-associated neurotoxicity syndrome. ^a^ No statistics were computed because Lymphopenia (days 1–28 post-infusion) was observed in all patients.

## Data Availability

The original contributions presented in this study are included in the article/Supplementary Materials. Further inquiries can be directed to the corresponding authors.

## Supplementary Materials

The following supporting information can be downloaded at: https://www.mdpi.com/article/10.3390/ijms27073113/s1.

## Abbreviations

The following abbreviations were used in this manuscript:

MM	Multiple myeloma
BCL	B cell lymphoma
CAR-T	Chimeric antigen receptor T cell
BCMA	B cell maturation antigen
R/R	Relapsed/Refractory
CR	Complete response
CMV	Cytomegalovirus
EBV	Epstein–Barr virus
ADV	Adenovirus
HBV	Hepatitis B virus
HCV	Hepatitis C virus
PCR	Polymerase chain reaction
rCMV	CMV reactivation
CS	Clinically significant
PFS	Progression free survival
OS	Overall survival
ECOG	Eastern Cooperative Oncology Group
CRP	C reactive protein
WBC	White blood cells
ANC	Absolute neutrophil count
ALC	Absolute lymphocytes count
IgG	Immunoglobulin G
IgM	Immunoglobulin M
IgA	Immunoglobulin A
CRS	Cytokine release syndrome
ICANS	Immune Effector Cell-Associated Neurotoxicity Syndrome
IMWG	International Myeloma Working Group
ASTCT	American Society for Transplantation and Cellular Therapy
EBMT	European Society for Blood and Marrow Transplantation
EHA	European Hematology Association
ASCT	autologous stem cell transplantation
Yescarta	axicabtagene ciloleucel
Kymriah	Tisagenlecleucel
Tecartus	brexucabtagene autoleucel
ICI	immune checkpoint inhibitors
Ab	antibody
