# Supplementary material for: Viral Reactivation in Multiple Myeloma Patients Receiving Anti-BCMA Chimeric Antigen Receptor T-Cell Therapy
Source: Int J Mol Sci. 2026 Mar 30;27(7):3113. doi: 10.3390/ijms27073113 (PMC13073535; doi:10.3390/ijms27073113)
Supplement: Supplementary file 1 [file ijms-4135778-supplementary.pdf › ijms-4135778-supplementary.pdf]

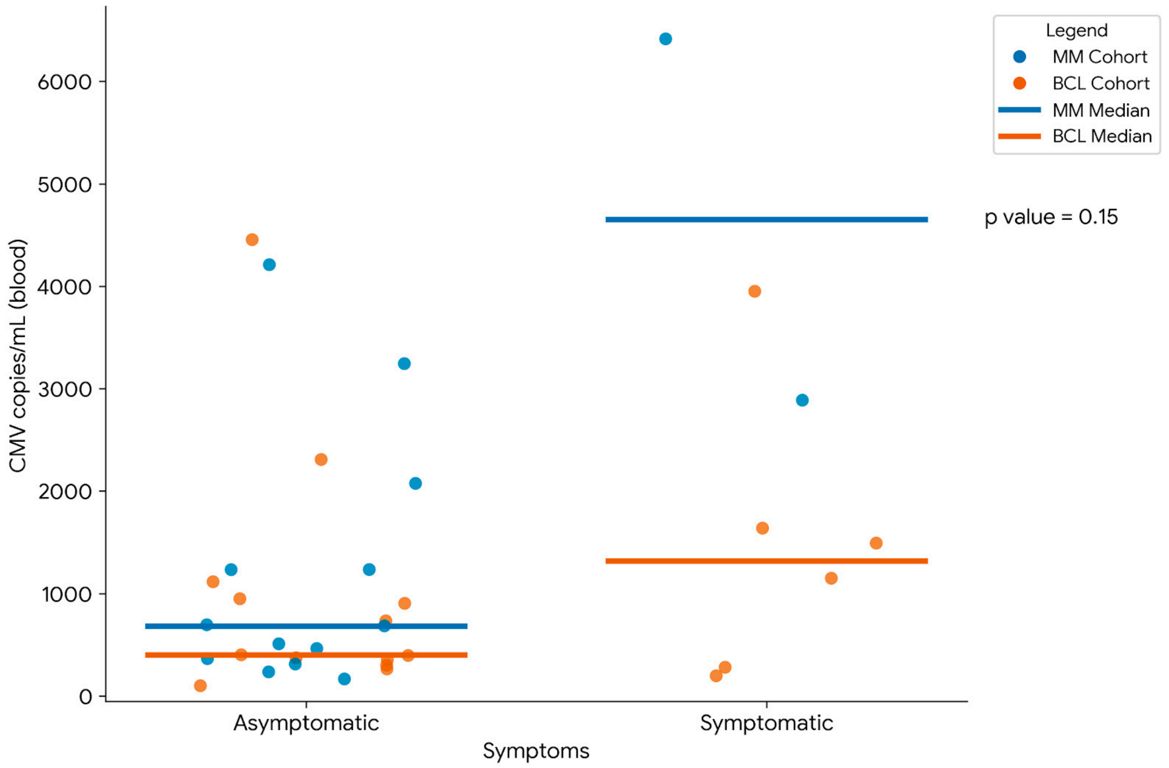

Supplementory Figure S1. CMV PCR viral load (copies/mL) in Asymptomatic and symptomatic (clinical significant) patients.
